# Exploring enablers and barriers to implementing the Transparency and Openness Promotion Guidelines: a theory-based survey of journal editors

**DOI:** 10.1098/rsos.221093

**Published:** 2023-02-01

**Authors:** Kevin Naaman, Sean Grant, Sina Kianersi, Lauren Supplee, Beate Henschel, Evan Mayo-Wilson

**Affiliations:** ^1^ School of Public Health, Indiana University-Bloomington, Bloomington, IN, USA; ^2^ School of Education, Indiana University-Bloomington, Bloomington, IN, USA; ^3^ HEDCO Institute for Evidence-Based Educational Practice, University of Oregon, Eugene, OR, USA; ^4^ Richard M. Fairbanks School of Public Health, Indiana University-Indianapolis, Indianapolis, IN, USA; ^5^ Brigham and Women's Hospital and Harvard Medical School, Boston, MA, USA; ^6^ William T. Grant Foundation, New York, NY, USA; ^7^ Gillings School of Global Public Health, University of North Carolina-Chapel Hill, Chapel Hill, NC, USA

**Keywords:** behaviour change, journal editors, reproducibility, open science, Top Guidelines, transparency

## Abstract

The Transparency and Openness Promotion (TOP) Guidelines provide a framework to help journals develop open science policies. Theories of behaviour change can guide understanding of why journals do (not) implement open science policies and the development of interventions to improve these policies. In this study, we used the Theoretical Domains Framework to survey 88 journal editors on their capability, opportunity and motivation to implement TOP. Likert-scale questions assessed editor support for TOP, and enablers and barriers to implementing TOP. A qualitative question asked editors to provide reflections on their ratings. Most participating editors supported adopting TOP at their journal (71%) and perceived other editors in their discipline to support adopting TOP (57%). Most editors (93%) agreed their roles include maintaining policies that reflect current best practices. However, most editors (74%) did not see implementing TOP as a high priority compared with other editorial responsibilities. Qualitative responses expressed structural barriers to implementing TOP (e.g. lack of time, resources and authority to implement changes) and varying support for TOP depending on study type, open science standard, and level of implementation. We discuss how these findings could inform the development of theoretically guided interventions to increase open science policies, procedures and practices.

## 1. Introduction

Journals in the behavioural, social and health sciences often publish articles with results that cannot be reproduced [1–6]. Irreproducibility and false findings in the published literature might be explained partly by common detrimental research practices associated with opaque and closed research workflows [7–13]. Journal policies (i.e. ‘instructions to authors') that promote transparent and open science could reduce these detrimental research practices [14–17].

### 1.1. The Transparency and Openness Promotion Guidelines

The Transparency and Openness Promotion (TOP) Guidelines are a prominent framework to help journals develop and implement clear policies regarding open science [18]. As described in box 1, TOP comprises eight standards on transparency (design and analysis reporting guidelines), reproducibility (data, code and materials sharing), prospective registration (study and analysis plan preregistration), and rewarding researchers for engaging in open science (conducting replications, and citing data, code and materials). Journals might not mention an open science practice or merely encourage authors to implement the open science practice in their journal policies (Level 0). Journals that adopt TOP can: require that authors disclose whether (or not) they used an open science practice (Level 1), require that authors use an open science practice (Level 2), or require that the journal verify the transparency and reproducibility of authors' research (Level 3). Thus, the lowest threshold for adopting TOP is implementing at least one standard at Level 1 (e.g. requiring authors to disclose whether or not their data are publicly available). Journal implementation of TOP standards is a target behaviour for many influential initiatives and organizations working to increase the credibility of published results [19–21].

Box 1. **The Transparency and Openness Promotion (TOP) Guidelines**

Standards

• **Citation Standards:** Citation of datasets in the text and reference sections of manuscripts
• **Data Transparency:** Public availability and sharing of datasets
• **Analytic Methods (Code) Transparency:** Public availability and sharing of analytical (statistical) code
• **Research Materials Transparency:** Public availability and sharing of other research materials
• **Design and Analysis Transparency:** Transparent reporting of study design and analysis
• **Study Preregistration:** Specification of study details prior to conducting the study
• **Analysis Plan Preregistration:** Specification of analytical details prior to conducting the study
• **Replication:** Encourages publication of replication studies

Levels of Implementation

• **Level 0** (**Not Implemented**)**:** The journal encourages an open science practice or says nothing about the open science practice.
• **Level 1 (Disclosure):** Published manuscripts disclose whether or not the study incorporated the open science practice.
• **Level 2 (Requirement):** A study must incorporate the open science practice for the manuscript to be published.
• **Level 3 (Verification):** The journal (or another independent third party) verifies that the study appropriately incorporated the open science practice according to journal standards.

Adapted from previously published grids [18].

Efforts to promote TOP implementation have seen mixed results. The Center for Open Science—which led the development and coordinates implementation of TOP—lists over 5000 signatories on their website (https://www.cos.io/initiatives/top-guidelines). Signatories include individual journals and societies, as well as large publishers of multiple journals, who have expressed ‘interest in the guidelines and commit to conducting a review within a year of the standards and levels of adoption'. This nominal approval of TOP is enabled by the growing proportion of scientists who practice open science [22] and support the specific practices in TOP, such as registration [23], replication [24], transparent reporting [25], data sharing [26–32], materials sharing [33] and code sharing [22,34,35]. In addition, journal editors increasingly support data sharing [33,36–41], and major funders are implementing requirements for data sharing [42–45] and study registration [46,47].

Becoming a TOP signatory does not always translate to implementation. In a database tracking TOP implementation, the modal journal does not implement any open science policies, and most standards in TOP are not implemented by most journals (https://www.topfactor.org/). Several independent assessments have also found low levels of TOP implementation [48–53]. Potential barriers include disinclinations toward prescriptive guidelines generally, disagreement with TOP specifically, scepticism about the outcomes of TOP implementation, time and effort required, and perceptions that TOP is not implemented or valued by peers [24,25,27,31,54–62]. Yet scant research has systematically investigated enablers and barriers to TOP implementation in a manner that would inform interventions to increase its uptake.

### 1.2. Using behaviour change theory to promote TOP implementation

**Figure 1.**
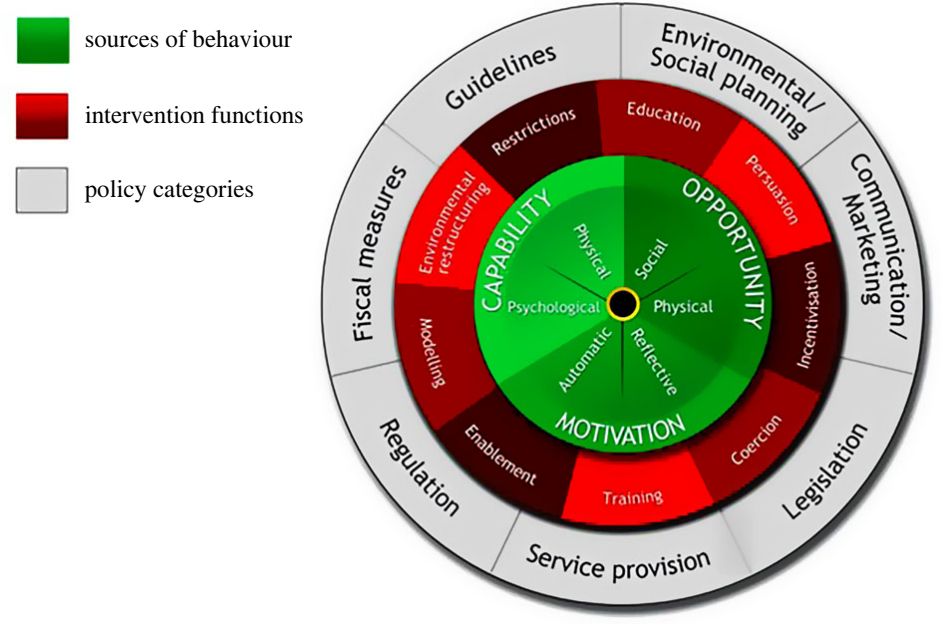
Behaviour Change Wheel. Reproduced under a CC BY 2.0 license from Michie *et al*. [67].

Because TOP implementation is a behaviour, theories of behaviour change can guide research to understand why journals do or do not have open science policies [63]. Intervention development should draw upon explicit theories and approaches for identifying hypothesized pathways from candidate intervention techniques to desired behaviour changes [64–66]. For example, the Behaviour Change Wheel (BCW) provides systematic guidance on developing behaviour change interventions, based on a broad range of multidisciplinary frameworks (figure 1) [67]. The BCW is centred on the ‘COM-B' theoretical model of behaviour, which posits that the likelihood an individual will engage in a behaviour is affected by their capability, opportunity and motivation to enact that behaviour. The COM-B maps onto the Theoretical Domains Framework (TDF), which divides capability, opportunity and motivation into 14 component theoretical constructs representing potential enablers and barriers to behaviour change [68]. Formative research can use the TDF to inform theoretically guided behaviour change interventions by identifying enablers of and barriers to behaviour change. Researchers can then use the BCW to link these enablers of and barriers to specific behaviour change techniques [69]. The TDF and BCW have been applied to a diverse range of behaviours, including researcher use of open science practices [70,71]; however, research has not yet used this approach to promote journal implementation of open science policies.

### 1.3. Objective

In this formative study, we sought to explore possible enablers and barriers to TOP adoption by asking editors to complete a survey that we developed using prominent theory and previous questionnaires from implementation science. We did not seek to test any prespecified hypotheses, and we did not register a protocol for the study.

## 2. Methods

We used the TDF to develop an online, mixed-methods questionnaire on enablers and barriers to journal implementation of TOP. The survey, invitations and reminders are available on Open Science Framework [72]. From 15 March 2021 to 26 April 2021, we surveyed editors of journals that publish influential intervention research about their (i) perceived and actual support for TOP and (ii) capability, opportunity and motivation to implement TOP. Our research data [73], code and materials (e.g. survey, emails) are available on Open Science Framework [74].

### 2.1. Sampling procedures

In a previous study, we identified 10 federal evidence clearinghouses that rate the quality of evidence concerning the effectiveness of social interventions [75]. We then identified 339 journals that published at least one intervention report that a clearinghouse used to give its highest rating for quality of evidence [76]. Most eligible journals were categorized in Journal Citation Reports as social sciences, psychiatry/psychology, clinical medicine or multidisciplinary [48]. For this study, we excluded one journal that ceased publication in 2020 and one journal for which we could not find any editor contact details.

We sent 337 editors an email invitation via Qualtrics (https://qualtrics.com/) to participate in our online survey. Our invitation email described the purpose of the study and included a unique link to the survey for each editor (electronic supplementary material). Our emails also informed editors that, upon completing the survey, they would be directed to individualized reports describing their journals' current implementation of TOP and changes they could make to increase implementation [77]. If an editor did not respond to the first invitation, we sent up to two reminder emails. Because emails sent through Qualtrics might be identified as ‘spam', we used a university email account to send reminders approximately one and two weeks after the first invitation. We found seven journals with an editor-in-chief on extended leave (e.g. sabbatical) or no longer affiliated with the journal; for these journals, we contacted the next editor listed on each journal's editorial board.

### 2.2. Data collection

We designed our questionnaire based on previous questionnaires from implementation science using the TDF [78–83]. The first page of the survey provided editors with a brief overview on the eight modular standards in TOP and their levels of implementation. On the next page, we presented questions for editors to rate on a five-point Likert scale ranging from ‘strongly disagree to ‘strongly agree’ (box 2). The first two questions asked whether editors support adopting TOP at their journal and whether other editors in their discipline support adopting TOP. We defined adopting TOP as implementing at least one of the eight open science standards at Level 1 (Disclosure) or higher. The remaining questions assessed enablers and barriers to implementing TOP based on 14 constructs in the TDF [68]. The final page asked editors for any feedback and reflections about their responses.

Box 2. **Survey Questions**

Part 1: Support for adoption of TOP Guidelines (Likert-Scale)

• **Actual support:** As editor, I support adoption of the TOP Guidelines at < insert *journal name* > .
• **Perceived support:** Other editors in my discipline support adoption of the TOP Guidelines at their respective journals.

Part 2: Enablers and barriers to implementing the TOP Guidelines (Likert-Scale)

• **Knowledge:** I am familiar with the content and objectives of the TOP Guidelines.
• **Cognitive and interpersonal skills (Skills)**: I have the necessary skills to adopt the TOP Guidelines at < insert *journal name* >.
• **Memory, attention and decision processes (Memory processes)**: When managing a manuscript at < insert *journal name*>, it is easy for me to remember the specific requirements in our ‘instructions to authors' that I am supposed to enforce.
• **Behavioural regulation**: I have a clear plan of how I could promote changes to ‘instructions to authors' at < insert *journal name*>, if I wanted to do so.
• **Social influences**: Colleagues whose opinion I value would approve of < insert *journal name* > adopting the TOP Guidelines.
• **Environmental context and resources (Environment)**: <insert *journal name* > has the necessary editorial systems and tools to adopt the TOP Guidelines.
• **Social/professional role and identity (Professional identity)**: It is part of my role as editor at < insert *journal name* > to maintain ‘instructions for authors' that reflect current best practices.
• **Beliefs about capabilities (Beliefs in capabilities)**: I am confident that, if I wanted, I would be capable of leading the adoption of the TOP Guidelines at < insert *journal name* > .
• **Optimism**: When < insert *journal name* > adopts new ‘instructions for authors', I usually expect positive outcomes.
• **Intentions**: I intend to promote the adoption of the TOP Guidelines at < insert *journal name* > in the next year.
• **Goals**: Compared with other editorial tasks, adopting the TOP Guidelines at < insert *journal name* > is a higher priority on my agenda.
• **Beliefs about consequences (Consequences)**: Adoption of the TOP Guidelines would benefit < insert *journal name* > .
• **Reinforcement**: Whenever I promote changes to the ‘instructions for authors' at < insert *journal name*>, I receive positive recognition from colleagues who are important to me.
• **Emotion**: I generally do not feel nervous or anxious about promoting adoption of the TOP Guidelines at < insert *journal name* > .

Part 3: Reflections (Qualitative)

• We welcome any reflections on your responses and feedback below.

### 2.3. Data analysis

We analysed the 16 Likert-scale questions by counting the number of responses in each of the response categories. We visualized these results using bar charts. We narratively combined percentages for ‘strongly agree' with ‘somewhat agree' and ‘strongly disagree’ with ‘somewhat disagree'. To explore potential sources of heterogeneity, we stratified the proportions of editors who agreed and disagreed with each item by whether their journals were not listed as TOP signatories on the Center for Open Science website [84]. To explore potential non-response bias, we examined whether TOP implementation [48] and bibliometric characteristics [85,86] differed between journals of participating and non-participating editors by comparing measures of central tendency and dispersion, visualizing density distributions and histograms, and conducting Welch two-sample *t*-tests. For data cleaning and visualizations, we used the tidyverse [87], Likert [88], ggpubr [89] and table1 [90] packages in RStudio 4.0.1 [91,92]. We cleaned and processed TOP implementation data and bibliometric characteristics using the pandas [93,94] and NumPy [95] packages in Python 3.7.6 [96]. Lastly, we analysed written reflections by grouping comments into shared topics and creating topic summaries [97].

## 3. Results

**Figure 2.**
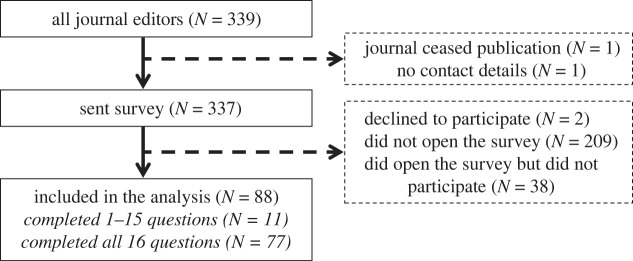
Flowchart of journal editor participation in our survey.

**Figure 3.**
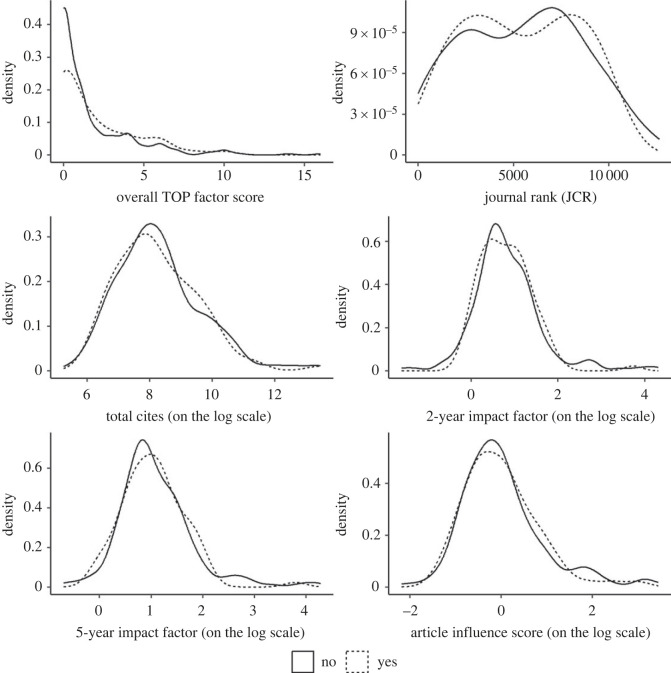
Journal TOP implementation and JCR rankings by survey participation status. The **TOP Factor** measures the extent that journals have implemented the TOP Guidelines into their editorial policies and has a range from 0 to 29. Our analyses of JCR metrics are based on the 2019 data. As defined by JCR [98], **Total Cites** is ‘the total number of times that a journal has been cited by all journals included in the database in the JCR year' (i.e. 2019). **2-year Impact Factor** is ‘defined as all citations to the journal in the current JCR year to items published in the previous 2 years, divided by the total number of scholarly items (these comprise articles, reviews and proceedings papers) published in the journal in the previous two years'. The **5-year Impact Factor** is ‘the average number of times articles from the journal published in the past five years have been cited in the JCR year. It is calculated by dividing the number of citations in the JCR year by the total number of articles in the five previous years'. The **Article Influence Score** ‘is calculated by multiplying the *Eigenfactor* Score by 0.01 and dividing by the number of articles in the journal, normalized as a fraction of all articles in all publications…The Eigenfactor Score calculation is based on the number of times articles from the journal published in the past five years have been cited in the JCR year, but it also considers which journals have contributed these citations so that highly cited journals will influence the network more than lesser cited journals’.

**Figure 4.**
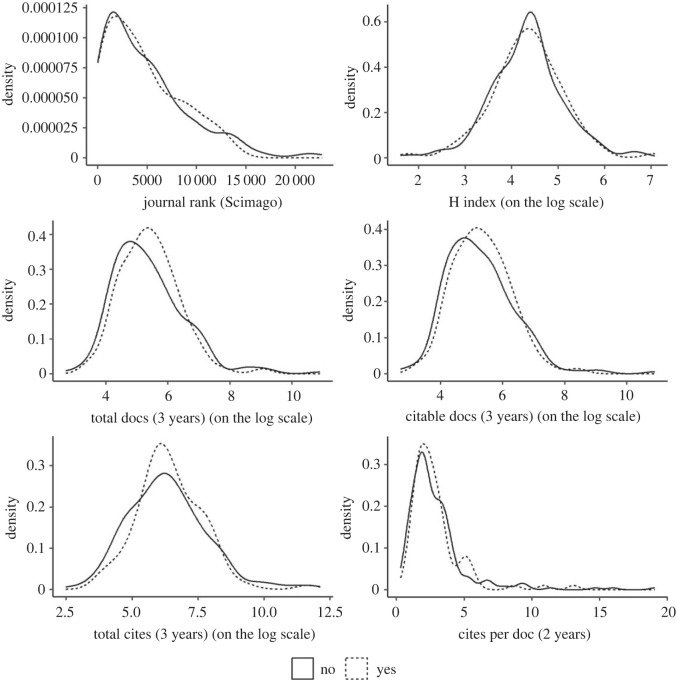
Journal SCImago rankings by survey participation status. Our analyses of SCImago metrics are based on the 2020 data. As defined by SCImago [99], **Journal Rank** is the ‘average number of weighted citations received in the selected year by the documents published in the selected journal in the three previous years'. **H Index** is ‘the journal's number of articles (*h*) that have received at least h citations'. **Total docs (3 years)** is the number of ‘published documents in the three previous years'. **Citable docs (3 years)** is the ‘number of citable documents published by a journal in the three previous years'. **Total cites (3 years)** is the ‘number of citations received in the selected year by a journal to the documents published in the three previous years'. **Cites per doc (2 years)** is the ‘average citations per document in a 2-year period. It is computed considering the number of citations received by a journal in the current year to the documents published in the two previous years'.

Of 337 eligible editors, we recruited 88 (26%) to participate in our survey (figure 2). Of invited editors, 62% (209/337) did not open the link, 11% (38/337) opened the survey but did not complete any questions, and 1% (2/337) declined to participate by emailing us. Of participating editors, 87% (77/88) answered all 16 Likert-scale questions. We did not identify evidence of non-response bias between participating and non-participating editors on TOP implementation or bibliometric characteristics of their journals based on our statistical analyses (S1) or inspections of visualized density distributions (figures 3 and 4; electronic supplementary material, figures S2–S7).

### 3.1. Quantitative findings

**Figure 5.**
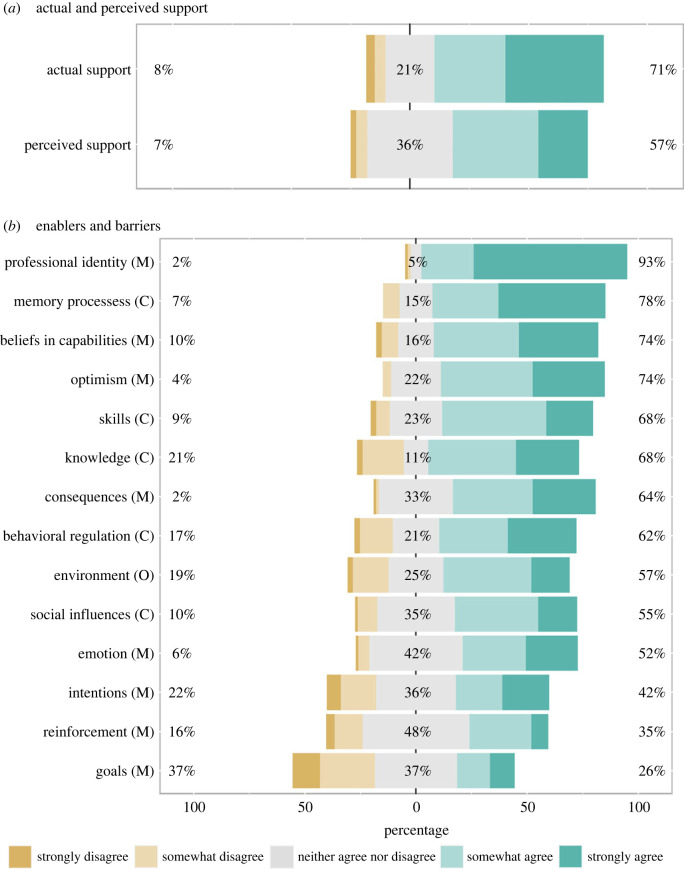
Results of quantitative rating questions. Theoretical Domains Framework sources of behaviour: C = Capability; O = Opportunity; M = Motivation. Number of responses per question ranged from 79 to 87. In Part (*b*), ‘agree' indicates that the source of behaviour is an enabler of TOP adoption and ‘disagree' that the source of behaviour is a barrier to TOP adoption. Professional identity = Social/professional role and identity. Memory processes = Memory, attention, and decision processes. Beliefs in capabilities = Beliefs about capabilities. Skills = Cognitive and interpersonal skills. Consequences = Beliefs about consequences. Environment = Environmental context and resources.

As shown in figure 5*a*, most participating editors support adopting TOP at their journals (71%; 62/87) and perceive that other editors in their discipline support adopting TOP at their journals (57%; 49/86). As shown in figure 5*b*, the degree to which editors perceived TDF domains as enablers or barriers varied (electronic supplementary material, tables S8 and S9 include the number of editors who responded to each item).

**Figure 6.**
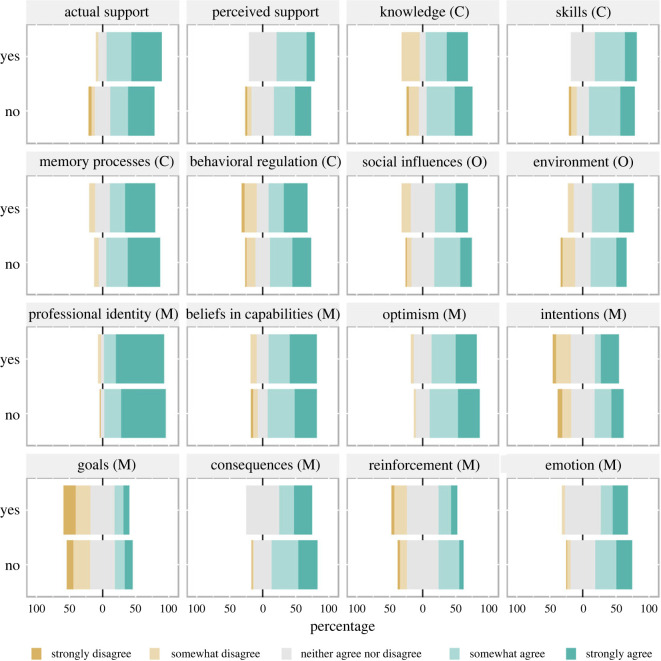
Quantitative Results Stratified by TOP Signatory Status. ‘Yes' indicates TOP signatory journals and ‘No' indicates non-signatories. ‘Agree' indicates that the source of behaviour is an enabler of TOP adoption and ‘disagree' that the source of behaviour is a barrier to TOP adoption. Theoretical Domains Framework sources of behaviour: C = Capability; O = Opportunity; M = Motivation. Number of responses per question ranged from 21 to 24 for TOP signatories and from 58 to 63 for non-signatories. The Center for Open Science stopped adding journal names to their list of signatories at the end of 2020. We collected data in March 2021, so our analysis of heterogeneity may have included more journals in the TOP signatory group if the Center for Open Science had continued updating their list of signatories. Professional identity = Social/professional role and identity. Memory processes = Memory, attention, and decision processes. Beliefs in capabilities = Beliefs about capabilities. Skills = Cognitive and interpersonal skills. Consequences = Beliefs about consequences. Environment = Environmental context and resources.

Most respondents were editors of journals that were not signatories of TOP (73%; 64/88). We did not identify important differences in support for TOP, or in perceptions of enablers and barriers, when comparing signatories and non-signatories (figure 6).

#### 3.1.1. Capability

Most editors agreed that they have the capability to implement TOP: 78% (63/81) believe it is easy to enforce journal policies once enacted, 68% (55/81) that they have both the skills and knowledge to implement TOP and 62% (50/81) that they have a clear plan of how they could promote changes to their journal policies if desired.

#### 3.1.2. Opportunity

Slightly more than half of editors agreed that they have the opportunity to implement TOP: 57% (46/81) believe that they have the necessary editorial systems and tools and 55% (44/80) that colleagues whose opinion they value would approve of them implementing TOP.

#### 3.1.3. Motivation

The majority of editors agreed that part of their role is to maintain journal policies that reflect current best practices (93%; 75/81). Most editors also had confidence in their ability to facilitate TOP implementation at their journal (74%; 60/81), expected positive outcomes from journal implementation of new policies (74%; 59/80), and believed that implementing TOP would benefit their journal (64%; 52/81). Slightly more than half (52%; 42/81) were not nervous or anxious about promoting TOP. However, 74% (60/81) did not see TOP as a high priority compared with other editorial tasks, 65% (51/79) do not receive positive recognition from colleagues who are important to them when changing journal policies, and 58% (47/81) did not intend to implement TOP in the next year.

### 3.2. Qualitative findings

We identified several topics in written reflections (electronic supplementary material, box S10), which we received from 19% (17/88) of editors.

#### 3.2.1. Overall support

Editors indicated overall support for open science policies and for TOP specifically, including editors of journals that had either already or planned to implement TOP: ‘We are in the process of announcing guidelines that include many of the TOP transparency guidelines'.

#### 3.2.2. Differences by context and discipline

Several editors indicated varying support for TOP depending on context and discipline: ‘The TOP guidelines try to be “one size fits all”. They do not’. For example, support for TOP can vary by the type of studies to which it is being applied: ‘The main challenge is that we are eclectic with respect to the types of studies we accept. I am 100% behind the adoption of TOP for trials, but that is not all that we do. Standards for other types of studies are less well-developed. That does not mean we shouldn't do it across study designs, but as an editor I cannot be vague about requirements'. In addition, support for TOP can vary by open science practice: ‘I agree with adopting some of the TOP guidelines but not with adopting all of them'. Support for TOP also can vary by level of implementation: ‘Being transparent about whether a particular article does this [use an open science practice] is an easy choice…[but] another issue to consider is that…there is always a risk that enough information can be gathered from different sources to identify individuals'.

#### 3.2.3. Enablers and barriers

Other topics provided elaboration on enablers and barriers examined in the questionnaire. For example, one editor explained why TOP is a low priority: ‘TOP guidelines are not a very high priority concern relative to daily priorities for running the journal…At this moment our focus has to be on daily operations and in the long run, having a diversity statement that contains measurable objectives. TOP was not even on my list until this survey'. Editors also expressed concerns about TOP implementation leading to increased demands, especially for more stringent levels of implementation: ‘moving to what TOP Guidelines define as Level 3 requires time and somehow a cultural change in our specific authors…This is something that has to be done gradually, introducing step by step new requirements for our authors'. Several editors also spoke to their organizational context, namely the role of the editorial board, sponsoring society and publisher: ‘we don't have the authority to implement these guidelines; it would go through other channels (the journal committee, the executive committee)'. The kind of influence that these other stakeholders have may vary by discipline, business model of the journal and publisher capacity: ‘We are changing publishers next year, which will make it much easier to adopt the guidelines'. Lastly, several editors said that they needed more knowledge about and skills in implementing TOP to consider it further: ‘I am new to much of this but think it potentially important and will look into it more’.

## 4. Discussion

We found most participating editors support implementing at least one of the eight open science standards at Level 1 or higher, and they perceive that other editors also support some level of adoption. Consistent with previous studies [22,54,57], editors perceive that peer support is lower than actual support, suggesting that editors might be unaware how much community norms have shifted recently in favour of TOP. Our survey also identified several potential enablers that are linked to theories of behaviour change. Most notably, editors perceive their roles to include maintaining policies that reflect current best practices. Other enablers included the ease of enforcing journal policies once enacted, editor confidence that they could facilitate TOP implementation at their journal if they so desired, and optimism that changes in journal policies lead to positive outcomes. Conversely, we identified several barriers to implementing TOP related to motivation, the most substantial being competing priorities, lack of intention to promote TOP and limited familiarity with TOP. Qualitative responses suggest that factors outside the direct control of journal editors—i.e. limited time, resources and authority to implement changes—may be important determinants of these motivational barriers. In addition to elaborating on these enablers and barriers examined in the questionnaire, qualitative responses also indicated that editor support can vary by study type, open science standard and level of implementation. We did not identify systematic differences in enablers and barriers based on TOP signatory status.

### 4.1. Theoretically informed interventions to promote TOP implementation

Our findings have implications for the development of interventions to increase open science using best practices for translational research [100]. Intervention development approaches like the BCW can be used to operationally define journal adoption of TOP as a targeted behaviour for intervention. Combined with the TDF, the BCW can then be used to identify intervention techniques that address enablers and barriers to increasing the prevalence of the target behaviour (i.e. TOP adoption) among the population of interest (i.e. journals) [64,65,101,102]. For example, our findings suggest motivation is an important barrier to TOP implementation, particularly what the TDF classifies as ‘reflexive motivation' represented by goals and professional identity. According to the BCW approach, techniques that target reflexive motivation might include working with editors to set goals for behaviours to be achieved (e.g. at least one TOP standard implemented at Level 1). Additionally, interventions might help editors identify positive outcomes of these behaviours (e.g. increased visibility of publicly available datasets). Journal progress toward these goals could then be reviewed periodically by examining changes in the desired behaviours and outcomes. As another example from the BCW approach, techniques like personalized feedback could provide journal editors with data on TOP implementation over time, drawing attention to discrepancies between current implementation with agreed goals and TOP implementation at peer journals. Based on our survey finding professional identity as a substantial enabler, this feedback could also instruct editors on how to implement TOP, offer solutions for overcoming factors that might impede TOP implementation, and provide contact details to groups that can provide practical support on implementing TOP (e.g. the Center for Open Science). In the light of qualitative findings that these motivational barriers are driven by factors outside of direct journal editor control, information on advocacy with journal publishers and societies might also be beneficial to include, as the power and resources to make changes to journal policies often depends on approval from these authorities. Specifically, the BCW approach suggests that official guidelines on open science standards endorsed by and support services offered by these authorities would support the aforementioned interventions [67].

Our findings also indicate that interventions to implement open science journal policies should target publishers and manuscript submission systems. That is, we found that many editors do not plan to implement open science policies, and they report time as an important barrier. In an analysis related to this survey, we previously found that 335 eligible journals were affiliated with 86 publishers and 33 manuscript submission systems, and the majority of journals used the submission systems ScholarOne and Editorial Manager [103]. Working with publishers to change default options in widely used submission systems could be a scalable approach to increasing TOP. Even in the absence of stringent policies, these changes could prompt authors to see open science practices as more normative. These changes would also allow submission systems to capture structured data about open science practices, which might facilitate indexing of transparency information (e.g. in databases such as PubMed), and support automated surveillance and meta-research concerning TOP [104]. If it saves time and effort, our results suggest that authors and editors might support such changes.

### 4.2. Limitations

Our study has several limitations. Firstly, because we did not ask detailed questions about support for each specific TOP standard, some editors found our questions to be overly broad, and thus difficult to answer. To encourage participation, we intentionally designed a brief questionnaire and used the lowest threshold possible for adopting TOP (i.e. Level 1 implementation of at least one open science standard). Although a more detailed survey might have further clarified specific enablers and barriers to implementing specific TOP standards, we might have received fewer complete responses. Secondly, our questionnaire has not undergone psychometric validation. Although we organized our narrative around the three components of the COM-B model, we did not assess their reliability, so we focused our interpretation on the individual items. Future iterations of this questionnaire could be compared with our results and with other studies that assessed determinants of open research practices [70].

Our results might generalize to journals like those that participated in this study. Eligible journals had to publish studies of social and behavioural interventions, so our results might not generalize to disciplines and journals that do not publish this type of research. If editors who support TOP were most likely to participate, then our results might overestimate actual support, and they might overestimate the difference between perceived and actual support. Evidence that non-participating editors did not open the survey—choosing to ignore our unsolicited email invitation—suggests that non-response bias could be limited. We also did not find evidence that current open science policies or journal characteristics differed between journals whose editors participated and those whose editors did not participate, further reducing concerns about non-response bias.

## 5. Conclusion

We found support for the TOP Guidelines among editors of journals publishing influential intervention research. Quantitative findings identified enablers and barriers to implementing TOP that are linked to domains and constructs from theories of behaviour change. Qualitative responses elaborated on quantitative findings and further indicated that editor support can vary by study type, open science standard and level of implementation. Our findings can be used to develop theoretically informed and scalable interventions that aim to facilitate journal implementation of open science policies. Based on the BCW approach, these interventions include goal setting, action planning, monitoring and feedback, and instruction on and support in implementing TOP.

## Data Availability

De-identified data, statistical code for generating results and our survey instruments are available on the Open Science Framework (https://osf.io/j6ykx/). Supplementary material is available online [105].
